# Effects of different doses of methylprednisolone on clinical outcomes in patients with severe community-acquired pneumonia: a study protocol for a randomized controlled trial

**DOI:** 10.1186/s13063-022-06404-8

**Published:** 2022-05-21

**Authors:** Shukun Hong, Hongye Wang, Jian Liu, Lujun Qiao

**Affiliations:** 1 Department of Intensive Care Unit, Shengli Oilfield Central Hospital, Dongying, China; 2 Department of Obstetrics and Gynecology, Shengli Oilfield Central Hospital, Dongying, China

**Keywords:** Methylprednisolone, Glucocorticoid, Dose, Severe community-acquired pneumonia, Randomized controlled trial, Protocol

## Abstract

**Background:**

The specific use of methylprednisolone in severe community-acquired pneumonia (SCAP) has not yet formed a consensus. It is not clear whether the clinical efficacy of methylprednisolone in SCAP is dose-dependent, and how to balance the best efficacy with the least complications. The aim of this study is to evaluate the efficacy and safety of different doses of methylprednisolone in the adjuvant treatment for patients with SCAP.

**Methods/design:**

This is a prospective, randomized, double-blind, parallel group, placebo-controlled trial to evaluate the efficacy and safety of different doses of methylprednisolone in the adjuvant treatment for patients with SCAP. Patients with diagnosed SCAP are randomized to the following four groups at a 1:1:1:1 ratio: group 1 (control group)—standard ICU patient care+100ml of normal saline once a day for 5 days; group 2—standard ICU patient care+40mg of methylprednisolone (dissolved in normal saline with a final volume of 100ml) once a day for 5 days; group 3—standard ICU patient care+80mg of methylprednisolone (dissolved in normal saline with a final volume of 100ml) once a day for 5 days; and group 4—standard ICU patient care+120mg of methylprednisolone (dissolved in normal saline with a final volume of 100ml) once a day for 5 days. The primary outcome is PaO_2_/FiO_2_ ratio at day 5 following randomization. The secondary outcomes are 28-day mortality, ventilator-free days at 28 days, mechanical ventilation duration at 28 days, endotracheal intubation rate, time for temperature recovery, duration of vasopressors use, serum CRP and interleukin-6 level at day 5 following randomization, hospital stay, frequency of nosocomial infections, gastrointestinal hemorrhage, and hyperglycemia.

**Discussion:**

The results of our study may find the optimal dose of glucocorticoid in the adjuvant treatment of SCAP and provide evidence-based proof for clinicians to treat patients with SCAP. Since coronavirus disease 2019 (COVID-19) also belongs to community-acquired pneumonia, perhaps the results of our study will help to determine the appropriate dose of methylprednisolone in COVID-19 treatment.

**Trial registration:**

Chinese Clinical Trial Registry ChiCTR2100045056. Registered on 4 April 2021.

## Background

Community-acquired pneumonia (CAP) is a common clinical inflammatory disease, which is mainly caused by pathogenic microorganism infection. In Europe and North America, the annual incidence of adult CAP is 5–11 per thousand adult population and increases gradually with age [1]. Accounting for about 10–20% of hospitalized CAP, patients with severe CAP (SCAP) often need to be admitted to intensive care unit (ICU) for fluid resuscitation, necessary mechanical ventilation and other support treatment, and the 30 day mortality rate of these patients is as high as 23–47% [2–4]. Such a high mortality is not only related to the infection of SCAP itself, but also may be associated with the excessive inflammatory reaction after infection. The latter can cause extensive damage of alveolar epithelial cells and capillary endothelial cells, leading to the decrease of pulmonary surfactant, lung compliance, and lung consolidation, and eventually lead to the occurrence of acute respiratory distress syndrome.

From the perspective of pathological mechanism, the systemic inflammatory response produced by SCAP is the consequence of self-destruction caused by over activation of body defense mechanism, rather than the direct damage caused by pathogenic microorganism infection. Therefore, the treatment should not be limited to the control of infection and supportive treatment for respiration and circulation, but should find a way to block or reduce the self-destruction caused by the excessive inflammatory reaction and prevent tissue and cell damage.

Glucocorticoid is widely used in the treatment of severe infection because of its anti-inflammatory effect. Many evidence-based studies have reported that early application of glucocorticoid can affect the development of SCAP, promote disease recovery, and may reduce the mortality [5–8]. However, there is no consensus on the specific use of glucocorticoid in SCAP (such as type, dose, course of treatment, etc.) [4, 9, 10], and different methods of application bring diverse clinical outcomes [11–19]. Mikami et al. [13] found that administration of 40 mg of prednisolone for 3 days in patients with SCAP could promote resolution of clinical symptoms and reduce the duration of intravenous antibiotic therapy. Li et al. [16] recruited patients with SCAP complicated with septic shock to receive 80 mg of methylprednisolone or placebo for 3 days. The results showed that methylprednisolone could significantly improve oxygenation index, reduce serum C-reactive protein (CRP) level, and shorten the time of temperature recovery, duration of vasopressors use, and hospital stay, without increasing the incidence of adverse events. The findings of Li et al. were similar to the reports from another trial [18] that adopted 120mg of methylprednisolone to treat with patients with SCAP for 7 days. However, Zhao et al. [19] observed that although the improved oxygenation and the decreased levels of CRP and angiotensin II were detected after a 7-day therapy with 80–160mg of methylprednisolone, the length of hospital stay and mortality did not change, and the incidence of superinfection was significantly increased.

To sum up, the adjuvant effect of methylprednisolone in SCAP tends to be clear. However, the specific use of methylprednisolone has not yet formed a consensus, which needs further clinical demonstration. It is not clear whether the clinical efficacy of methylprednisolone in SCAP is dose-dependent, and how to balance the best efficacy with the least complications. As far as we know, no such research has been carried out.

The purpose of this superiority trial is to evaluate the efficacy and safety of different doses of methylprednisolone in the adjuvant treatment for patients with SCAP.

## Materials/design

### Setting

This prospective, randomized, double-blind, parallel group (1:1:1:1 ratio), placebo-controlled trial is to be conducted in Shengli Oilfield Central Hospital Affiliated to Binzhou Medical College. The hospital is a 1870-bed teaching hospital and located in the city of Dongying, Shandong province, China, which serves a population of about 2,170,000 people. The study will be carried out in three departments, including the ICU, the emergency ICU, and the respiratory ICU.

### Participants

All patients admitted to ICUs will be screened and evaluated on the day of admission. Patients who meet the inclusion criteria and do not meet the exclusion criteria will be invited to participate in the study.

Inclusion criteria:

1. Over 18 years of age at the time of diagnosis
2. Written informed consent is signed by the subject or the authorized representative
3. Patient diagnosed with SCAP according to the guidelines for the diagnosis and treatment of community-acquired pneumonia in Chinese adults (2016 Edition), formulated by respiratory branch of Chinese Medical Association. If a patient simultaneously meets the diagnostic criteria of CAP (shown in Table 1) and one of the following major criteria or more than three minor criteria, he/she can be diagnosed as SCAP

**Table 1:** Clinical diagnostic criteria of CAP

1. Onset of illness in the community	
2. Clinical manifestations	
• Recent cough, expectoration, or the aggravation of symptoms of original respiratory diseases, with or without purulent sputum, chest pain, dyspnea, and hemoptysis	
• Fever	
• Signs of pulmonary consolidation and/or moist rales were presented	
• Peripheral white blood cell counts > 10×10^9^ / L or < 4×10^9^ / L, with or without left shift of nucleus	
3. New patchy infiltrations, pulmonary consolidations, “ground-glass,” or interstitial changes are shown in chest imaging, with or without pleural effusion.	

Clinical diagnosis of CAP can be established after meeting items 1, 3, and any one of the item 2, and excluding pulmonary tuberculosis, pulmonary tumor, noninfectious pulmonary interstitial disease, pulmonary edema, atelectasis, pulmonary embolism, pulmonary eosinophilic infiltration, and pulmonary vasculitis

Major criteria:

- Invasive mechanical ventilation
- Septic shock with the need for vasopressors

Minor criteria:

- Respiratory rate ≥30 breaths/min
- PaO_2_/FiO_2_ ratio≤250 mmHg (1mmHg= 0.133kPa)
- Multilobar infiltrates
- Confusion/disorientation
- Blood urea nitrogen level≥7.14mmol/L
- Hypotension (systolic blood pressure< 90mmHg) requiring aggressive fluid resuscitation

Exclusion criteria:

1. Under 18 years of age at the time of diagnosis
2. Long-term use of glucocorticoids due to various underlying diseases (course of treatment ≥ 1 month)
3. Known allergy to glucocorticoids
4. Contraindication
  Systemic fungal infection
  Severe mental illness and history of severe mental illness
  Active peptic ulcer
  Recent gastrointestinal anastomosis
  Uncontrolled diabetes mellitus
  Uncontrolled varicella, measles and tuberculosis infection
5. Unwilling to receive positive therapy
6. Pregnant or breastfeeding women

### Randomization

Randomization will be based on a 1:1:1:1 allocation of pre-numbered boxes containing dosing units with methylprednisolone and normal saline and will be carried out by one of the investigators who will not participate in the inclusion of patients or in the delivery of medication. The allocation sequence will be computer-generated by a statistical expert and kept in a safe at the hospital pharmacy throughout the course of the study.

### Blinding (masking)

This trial is designed as a double-blind study to avoid introducing bias in the outcome assessment. The participants, investigators, care providers, and outcome assessors will be blinded to treatment allocation. Only pharmacists or nurses in the trial who are responsible for the randomization, separation, and blinding of the investigational drug will know the unblinded information. According to the allocation sequence, the pharmacists who do not participate in the outcome assessment will determine the investigational agent that each subject must receive. The agent solution that can be directly infused will be prepared by special nurses.

Early breaking of blind, if necessary, may be requested by the principal investigator and must occur in writing. The request may be motivated by emergency medical issues and/or legal and/or regulatory requirements. The principal investigator must document in writing the reasons for breaking the blind.

### Design

Patients with diagnosed SCAP are randomized to the following four groups at a 1:1:1:1 ratio:

1. Group 1 (control group): standard ICU patient care+100ml of normal saline once a day for 5 days. Standard ICU patient care involves ventilatory, hemodynamic, antimicrobial, nutritional, and other supports according to the guidelines for the diagnosis and treatment of community-acquired pneumonia in Chinese adults (2016 Edition). At present, there are no relevant guidelines to recommend methylprednisolone as a therapeutic drug for SCAP, which indicates that a consensus has not been reached and disputes still exist. Thus the placebo control group is still needed.
2. Group 2: standard ICU patient care+40mg of methylprednisolone (dissolved in normal saline with a final volume of 100ml) once a day for 5 days.
3. Group 3: standard ICU patient care+80mg of methylprednisolone (dissolved in normal saline with a final volume of 100ml) once a day for 5 days.
4. Group 4: standard ICU patient care+120mg of methylprednisolone (dissolved in normal saline with a final volume of 100ml) once a day for 5 days.

**Fig. 1:**
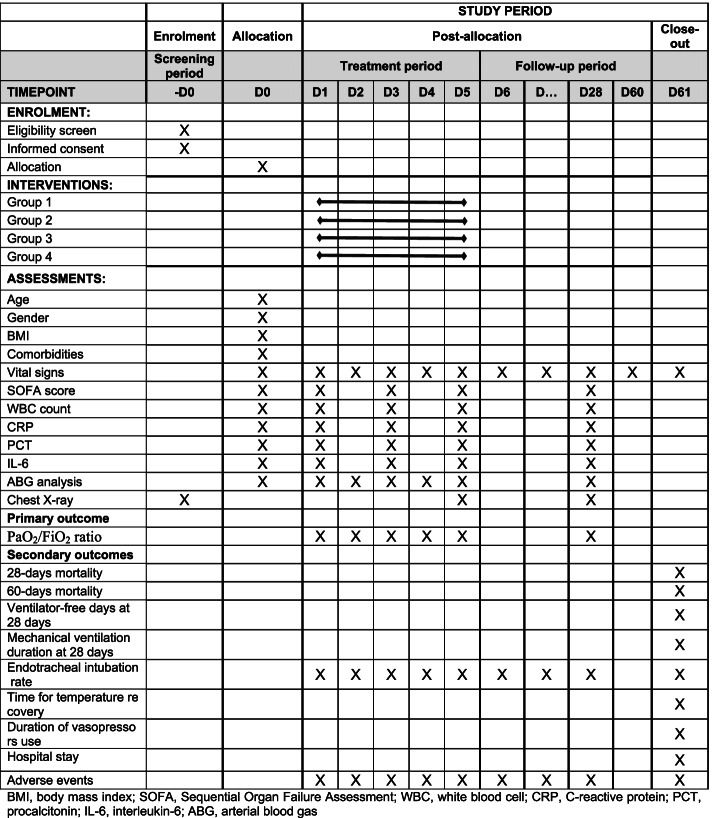
The schedule of enrolment, interventions, and assessments

Researchers will explain the research procedures in detail to the subject or agent to obtain the informed consent and will evaluate intervention adherence during each visit. If a scheduled visit is delayed or canceled, the research team will contact the subject immediately. No concomitant care or intervention is prohibited during the study. The trial is planned to be followed up for 28 days after randomization. The schedule of enrolment, interventions, and assessments is shown in Fig. 1.

### Primary outcome

- PaO_2_/FiO_2_ ratio at day 5 following randomization

### Secondary outcomes

- Twenty-eight-day mortality (death proportion within 28 days of randomization)
- Sixty-day mortality (death proportion within 60 days of randomization)
- Ventilator-free days at 28 days (the number of days free from mechanical ventilator support in the first 28 days following randomization)
- Mechanical ventilation duration at 28 days (the number of days during mechanical ventilation within 28 days of randomization).
- Endotracheal intubation rate (the proportion of patients requiring endotracheal intubation within 28 days of randomization)
- Time for temperature recovery (the number of days required for body temperature to return to normal)
- Duration of vasopressors use (the number of days required for continuous use of vasopressors)
- Serum CRP and interleukin-6 level at day 5 following randomization
- Hospital stay
- Frequency of nosocomial infections (the number of ventilator-associated pneumonia, blood stream infection or candidemia within 28 days of randomization)
- Gastrointestinal hemorrhage (the proportion of gastrointestinal bleeding patients requiring additional medication, blood transfusion, or surgical intervention within 28 days of randomization)
- Hyperglycemia (the proportion of patients with hyperglycemia requiring insulin injection within 28 days of randomization)

All adverse events and other unintended effects of trial interventions or trial conduct will be collected and documented, regardless of severity. Participants who suffer harm from these events will be compensated accordingly.

### Sample size

On the basis of previous study [12], it is calculated by Tukey-Kramer test that 220 participants will be required (55 in each group) to detect a difference of 50mmHg in the PaO_2_/FiO_2_ ratio between groups, assuming a standard deviation (SD) of 50mmHg in the control group (80% power, *α*= 0.05). If the follow-up rate is assumed to be 90%, 244 participants with 61 in each group will be needed. Sample size calculation was performed by PASS 11 software.

### Statistical analysis

We will show *n* (%) for categorical variables and median (interquartile range) for continuous variables with non-normal distribution or mean (SD) for those with normal distribution. Data for the primary and secondary outcome will be analyzed on intention-to-treat analysis, and missing data will be handled by multiple imputation method. If variances are equal, comparison of continuous variables will be evaluated by one-way analysis of variance, followed by the least significance difference procedure; otherwise, the Games-Howell procedure will be followed. The *χ*^2^ test followed by the Bonferroni method will be used to evaluate the comparison of categorical variables. Subgroup analysis will be used when appropriate. SPSS 18.0 software will be employed for statistical analysis. Significance level is set at 0.05.

### Data collection and management

Our study will use printed case report form (CRF) to collect the original data and will establish an electronic clinical research database to record all the information from CRF. We will use Epidata 3.1 software for dual data input and data proofreading. During the study, medical staff who did not participate in the study will supervise the trial. They will access the database to monitor all aspects of the study, including adherence to the study protocol, protection of participants, and accuracy of study data.

### Trial supervision

The data monitoring committee is established by the medical ethics committee and the Department of Science and Education of Shengli Oilfield Central hospital. According to the data review during the trial, the committee can make suggestions such as modifying the protocol and continuing or stopping the trial. Any important protocol modifications will be communicated to the relevant parties by the committee to ensure the consistency of the protocol. The datasets analyzed during the present study are available from the corresponding author on reasonable request.

### Confidentiality

We will only collect personal information of patients when necessary to assess the efficacy and safety of interventions. When dealing with such information, we will strictly abide by the laws on privacy protection and confidentiality. Paper documents involving patient information (including personal identity information and signed informed consent) will be stored in a locked filing cabinet. Similar electronic data will be stored in a password-protected computer.

### Clinical trial registration

The trial was registered under the registration number ChiCTR2100045056 on 4 April 2021 (http://www.chictr.org.cn/showproj.aspx?proj=121414). The study protocol was approved by the institutional medical ethics committee (approved No. of ethic committee: Q/ZXYY--ZY--YWB--LL201923).

## Discussion

Current reports have shown that glucocorticoids are effective in the treatment of coronavirus disease 2019 (COVID-19) [20, 21], and the latest meta-analysis [22] also shows that glucocorticoids are beneficial to acute respiratory distress syndrome caused by various diseases. Nevertheless, the specific types and doses of glucocorticoids in clinical application are uncertain. To contribute to this field of literature, our study was designed to address the question that whether different doses of methylprednisolone have an impact on the clinical outcomes of patients with SCAP.

At present, the timing of the use of glucocorticoids in the treatment of SCAP is still unclear. Theoretically, early SCAP is generally accompanied by systemic high inflammatory response, which can cause organ function damage, complicate the disease, and prolong the hospital stay. Glucocorticoids are classic anti-inflammatory drugs. In this sense, its early application in SCAP is reasonable. Moreover, previous studies of SCAP initiated glucocorticoid therapy mostly in the early stage of the disease [11–14]. It is beneficial to SCAP patients if glucocorticoid treatment is started after the diagnosis of SCAP [11–13] or within 36 h after admission [14]. Conversely, it has been reported that starting methylprednisolone treatment more than 1 week after the onset of acute respiratory distress syndrome closely related to SCAP does not bring benefits, while treatment more than 2 weeks after the onset may increase the risk of death [23]. This is why we chose to use glucocorticoid therapy in the early stage of SCAP.

Since patients with SCAP may need respiratory and/or circulatory support at any time, we believe that such patients should not be admitted to the general ward, but should be transferred to ICU for comprehensive treatment. Besides, it is necessary to evaluate whether there are contraindications before giving glucocorticoid therapy to these patients, and to pay attention to the occurrence of related complications (such as hyperglycemia, gastrointestinal hemorrhage, and secondary infection) during the treatment. Based on these considerations, we suggest that the prescription of glucocorticoids for SCAP patients should be determined by experienced clinicians from ICU.

From the perspective of molecular mechanism, glucocorticoid is a kind of liposoluble steroid hormone, which diffuses into the cytoplasm through cell membrane and plays a role by binding with glucocorticoid receptor (GR). There are four subtypes of human GR, including GRα, GRβ, GRγ, and GRδ. GRα is found in the cytoplasm of cells and is a classical receptor protein that can form complex with glucocorticoid. After hormone binding, GRα can activate gene transcription by interacting as a homodimer with glucocorticoid responsive elements (GREs) located in the promoter regions of target genes and inhibit gene transcription by interacting with negative GREs [24]. GRβ cannot bind glucocorticoid due to its altered ligand-binding domain, and although it can bind GRE, it cannot activate transcription of GRE [25]. However, many studies have found that the increase of GRβ expression is related to glucocorticoid resistance [26–28]. The molecular basis for the dominant-negative activity of GRβ appears to lie on the formation of GRα-GRβ heterodimer, which would hinder the formation of transcriptionally active GRα homodimer [25, 29]. In our study, different doses of glucocorticoid will be used in the adjuvant treatment of SCAP, which may have different effects on the expression of GRα and GRβ, resulting in different clinical outcomes. Unfortunately, there is no experiment on receptor expression in this study protocol. In the future, however, we plan to carry out corresponding research in animal experiments first.

The results of our study may find the optimal dose of glucocorticoid in the adjuvant treatment of SCAP and provide evidence-based proof for clinicians to treat patients with SCAP. In the context of the fact that COVID-19 epidemic is still spreading worldwide, many studies have shown that methylprednisolone is effective in treating severe COVID-19 patients [30, 31]. However, the specific dose of methylprednisolone remains unclear. Since COVID-19 also belongs to community-acquired pneumonia, perhaps the results of our study will help to determine the appropriate dose of methylprednisolone in COVID-19 treatment.

It is worth noting that since the diagnostic criteria of SCAP used in this study are formulated by the respiratory branch of Chinese Medical Association, whether the results of this study can be popularized in the world still needs further verification.

### Trial status

The trial was registered under the registration number ChiCTR2100045056 on 4 April 2021 (http://www.chictr.org.cn/showproj.aspx?proj=121414). The study protocol was approved by the institutional medical ethics committee (approved No. of ethic committee: Q/ZXYY--ZY--YWB--LL201923). Protocol version 3.0 date: 5 April 2021. The trial is already in the recruitment phase. Recruitment began 17 April 2021 and is anticipated to be complete by the end of December 2022. The final results will be reported in peer-reviewed publications after trial completion.

## Data Availability

The datasets analyzed during the present study are available from the corresponding author on reasonable request.

## Abbreviations

CAP: Community-acquired pneumonia
COVID-19: Coronavirus disease 2019
CRF: Case report form
CRP: C-reactive protein
GR: Glucocorticoid receptor
GREs: Glucocorticoid responsive elements
ICU: Intensive care unit
SCAP: Severe CAP
SD: Standard deviation
